# Identify the direct and indirect impacts of the community built environment on the health of older adults

**DOI:** 10.3389/fpubh.2025.1478337

**Published:** 2025-03-31

**Authors:** Jinsu Yang, Yuming Shang, Fengxiao Cao, Huaze Ying, Yu Luo

**Affiliations:** School of Architecture and Urban Planning, Fujian University of Technology, Fuzhou, China

**Keywords:** the community built environment, indirect impacts and direct impacts, structural equation modeling, older adults, health outcomes

## Abstract

**Background:**

The global ageing population is increasing. As their physical functions deteriorate, older adults face not only physical health challenges but also mental health issues. Enhancing the health status of older adults is imperative to improve their quality of life. However, research on the health status of older adults living in the community is limited, and the association between the built environment and daily activities remains largely unexplored.

**Objective:**

This study aimed to utilize structural equation modeling to (1) explore the interrelationships between the community built environment, daily activities of older adults, and their health, and (2) examine the interrelationships among their correlates.

**Methods:**

For data collection, this study administered structured questionnaires to 494 community-dwelling older adults across ten representative urban communities in Fuzhou, China. The questionnaire comprised four validated sections: demographic characteristics, perceived community built environment features, daily activity and health outcomes. Data analysis employed structural equation modeling (SEM) using AMOS 27.0, with SPSS 27.0 for preliminary analyses, to examine both direct effects of built environment on health outcomes and indirect effects mediated through daily activities.

**Results:**

Structural equation modeling revealed three pathways: Path 1 (community built environment →health of older adults), Path 2 (community built environment → daily exercise for older adults), and Path 3 (daily exercise for older adults →health of older adults). All three pathways were supported, indicating interaction among the factors.

**Conclusion:**

The health status of older adults is influenced by their living environment and daily activities. An improved community built environment can enhance health status among older adults. Furthermore, daily activities serve as partial mediators between community built environments and health outcomes. Our methodology and findings offer valuable insights for optimizing community built environments to promote the health of older adults.

## 1 Introduction

Population aging has emerged as a critical global public health issue and a shared challenge for all countries and regions in the 21st century (1). According to the National Bureau of Statistics of China, the country experienced its first instance of negative population growth in 2022, signifying its official entry into an era of sustained population decline. This demographic shift is accompanied by an increasing degree of aging, exacerbating associated social and public health concerns.

As physiological functions naturally decline with age, health challenges have become a central focus of aging-related issues in China (2, 3). Given that the majority of older adults’ daily activities occur within their residential communities, the built environment of these communities profoundly influences not only their activities but also their overall health status (4). Therefore, investigating the relationship between community built environments and the health of older adults is essential for developing strategies to enhance their well-being.

The World Health Organization (WHO) defines health as a state of complete physical, mental, and social well-being, rather than merely the absence of disease or infirmity (5). Existing research underscores the significant impact of community built environments and daily activities on both the physical and mental health of older residents (6–9). Accordingly, this study conceptualizes health as encompassing both physical and mental health.

Numerous studies have demonstrated that the community built environment significantly affects the health outcomes of older adults (10, 11). First, the high accessibility of community facilities—including recreational spaces (12), sports infrastructures (13), health education resources (14), medical service centers (15), older adult care institutions (16), supermarkets (17)—is crucial for promoting daily activity engagement among older adults. This accessibility effectively encourages older adults to engage in daily activities, thereby improving their overall health. Second, well—connected sidewalks and good crossing facilities facilitate active travel, while a convenient public transportation system augments the propensity of older individuals to engage in outdoor activities, thereby exerting a beneficial influence on their physical and mental health (4, 11, 18). Third, the safety of community spatial is crucial for ensuring the well-being of older adults. Safety of the transportation environment (19), pavement quality (20, 21) and noise management (22) in which older adults live can have an impact on their health. Fourth, the safety of community facilities is one of the core needs of older adults, as it is closely linked to their physical and mental health. Comprehensive security measures, such as barrier-free facilities and surveillance systems, further enhance psychological comfort, encourage outdoor activities, and improve overall physical and mental health (23–25). Fifth, exposure to natural landscapes provides considerable health benefits for older adults. Green spaces with high visibility (26, 27), the usual visual corridors, rich blue-green spaces (28, 29), interactive landscape elements (30), and high tree canopy cover all contribute to stress reduction and better health (31). Finally, the comfort of community site spatial environments influences travel behavior and, consequently, health outcomes. Clean and well-maintained streets with street-side resting places and shelters significantly increase older people’s willingness to walk outdoors, which is essential for physical and mental health (12, 23, 28) (Table 1).

**Table 1:** Summary of existing research.

Community built environment dimension	Elements	Specific impacts on health	Related research literature
Facility accessibility	Cultural facilities	Accessibility to recreational spaces helps slow the decline of physical functions.	Liu et al. (12)
	Sports facilities	Accessibility to sports infrastructures encourages older adults to actively participate in physical activities.	Xiao et al. (13)
	Education facilities	Accessibility to health education resources increases health knowledge and awareness.	Andersen et al. (14)
	Healthcare facilities	Accessibility to medical service centers provides essential care and support for the older adult, thereby enhancing their sense of well-being and fulfillment.	Zhao et al. (15)
	Care facilities	Accessibility to older adult care institutions, such as daycare centers and senior cafeterias, encourages older adults to leave their homes and engage in social activities.	Yafei et al. (16)
	Commercial services	Accessibility to supermarkets positively impacts the mental health of older adults.	Barnett et al. (17)
Mobility convenience	Pedestrian network connectivity	Areas with better road connectivity have better heart and respiratory health for older adults.	Niculita-Hirzel et al. (11)
	Pedestrian crossing accessibility	Well—designed footpaths and accessible street crossings are acknowledged as crucial elements in facilitating walking and daily activities among older adults.	Niculita-Hirzel et al. (11)
	Public transit accessibility	Transit route density positively correlated with older adults’ active travel.	Zhang et al. (18)
Spatial environment safety	Safety of the transportation environment	Road safety directly influences quality of life, serving as a critical safeguard for physical health	Shrivastava et al. (19)
	Pavement quality	Well-maintained sidewalks with adequate transportation infrastructure and sanitation facilities not only support physical health but also alleviate psychological stress, thereby fostering mental health.	Sallis et al. (20) and Anrooij et al. (21)
	Noise management	Older adults prefer natural acoustic environments, noise pollution reduces both their willingness to travel and their overall health outcomes	Wang and Kang (22)
Facility layout safety	Completeness of barrier-free facilities	Preparing the community built environment for aging with assistive devices (e.g., the presence of crosswalks, and paved or leveled walking paths) is important to promote independence and wellness.	Rosenberg et al. (23)
	Comprehensiveness of security monitoring facilities	Well-established security measures can promote health.	Liu et al. (24) and Shouyi et al. (25)
Landscape environment comfort	Aesthetic and recreational appeal of landscape features	Interaction with landscape elements can reduce stress.	Hassan and Deshun (30)
	Visual richness of landscape design	Blue-green spaces contribute to mental restoration.	Grey et al. (29) and Yaoqiong and Zhenwei (28)
	Unobstructed clarity of landscape visual corridors	Features such as visual corridors contribute to relaxation.	Grey et al. (29) and Yaoqiong and Zhenwei (28)
	Proportion of greenery in the visual field	Outdoor spaces with high green visibility alleviate the adverse effects of high-density urban environments, thereby improving mental health and life satisfaction.	Padeiro et al. (26) and Pan et al. (27)
	Tree canopy shading ratio	Tree canopy coverage is associated with better health outcomes.	Leigh and Leigh (31)
Site spatial comfort	Cleanliness of streets	Clean and well-maintained streets encourage walking and outdoor exercise, which are critical for physical and mental health.	Liu et al. (12) and Yaoqiong and Zhenwei (28)
	Accessibility of walking rest facilities	Providing on-street resting places and shelters is important for the health of older people.	Rosenberg et al. (23)
	Convenience of rain and sun protection facilities	Features such as shaded areas, rain shelters, and accessible seating significantly increase older adults’ willingness to walk outdoors.	Liu et al. (12) and Yaoqiong and Zhenwei (28)

Walking and outdoor exercise are two important forms of daily activities for older adults, with profound benefits for their health. Walking, as the most common activity among older adults, offers numerous physical and mental health benefits (32). Regular outdoor activities help prevent functional decline and promote mental health (33–35). Aerobic exercise, in particular, has been shown to mitigate mild cognitive impairment and delay cognitive deterioration (36, 37). The positive relationship between daily activities and health outcomes in older adults is influenced by factors such as activity frequency, duration, and type (38, 39).

In summary, given the significant aging population in China and the high prevalence of health issues among older adults—combined with their substantial reliance on community built environments for daily activities—there is a critical need to address the activity-related needs of older adults to enhance their health and well-being. This enhancement is essential for improving quality of life and life satisfaction among older adults.

Although prior studies have examined the link between community environments and older adults’ health, few have adequately explored the relationship between community built environments, older adults’ daily activities, and health outcomes. Thus, this paper introduces daily activities as a mediating variable and employs a structural equation model to investigate the interrelationships among the community built environment, daily activities, and health in older adults. The goal is to identify pathways and mechanisms within these relationships to inform strategic recommendations for community planning and development. The aim of this study is to propose evidence-based decision-making strategies for community planning updates by analyzing the pathways and mechanisms of interactions between variables, thereby promoting the development of age-friendly health communities.

## 2 Materials and methods

This cross-sectional study employed structural equation modeling (SEM) to analyze data from community-dwelling older adults in Fuzhou, China. The research methodology employed SEM as the primary analytical framework—a sophisticated multivariate statistical technique that integrates confirmatory factor analysis with path analysis to elucidate relationships between observed indicators and latent constructs.

The analytical protocol proceeded through sequential phases of theoretical model construction, systematic data collection, and statistical model fitting. SEM facilitated the simultaneous examination of direct pathways (community built environment → health outcomes) and indirect pathways mediated through daily activities, while accounting for measurement error inherent in psychosocial constructs. This methodological approach enabled precise quantification of parameter estimates for each hypothesized relationship, thereby illuminating the relative magnitude of environmental factors influencing older adults’ health outcomes and the mediating mechanisms through which these effects manifest.

### 2.1 Research objectives and hypotheses

**Figure 1:**
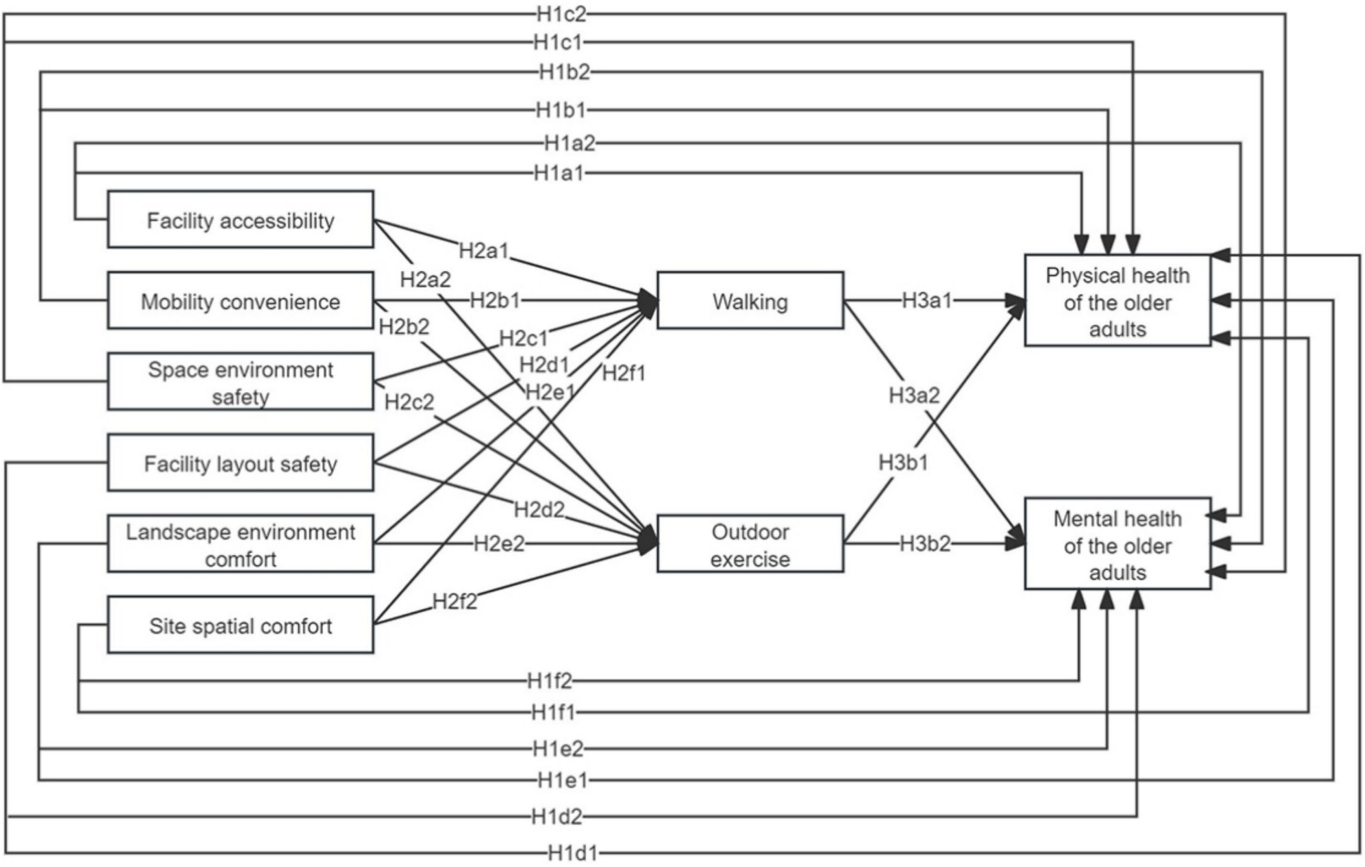
Diagram of the hypothetical model.

This study provides a thorough review and synthesis of existing literature, organizing the community built environment into six key dimensions: facility accessibility, mobility convenience, spatial environment safety, facility layout safety, landscape environment comfort, and site spatial comfort. Additionally, the daily activities of older adult participants are classified into two primary types: walking and outdoor exercise. Following the ten health standards established by the World Health Organization, older adult health is divided into two categories: physical and mental health. Prior research indicates that the community built environment significantly impacts both the daily activities and overall health of older adults. Furthermore, these daily activities exhibit reciprocal effects on health outcomes. Based on these insights, three hypotheses (H1, H2, and H3) are proposed (Figure 1).

H1: The community built environment has a significant direct positive impact on the health of older adults. H1a1 refers to the positive impact of facility accessibility on physical health, H1a2 refers to the positive impact of facility accessibility on mental health. H1b1 refers to the positive impact of mobility convenience on physical health, H1b2 refers to the positive impact of mobility convenience on mental health. H1c1 refers to the positive impact of spatial environmental safety on physical health, and H1c2 refers to the positive impact of spatial environment safety on mental health. H1d1 refers to the positive impact of facility layout safety on physical health, and H1d2 refers to the positive impact of facility layout safety on mental health. H1e1 refers to the positive impact of landscape environment comfort on physical health, and H1e2 refers to the positive impact of landscape environment comfort on mental health. H1f1 refers to the positive impact of site spatial comfort on physical health, and H1f2 refers to the positive impact of site spatial comfort on mental health.

H2: The community built environment has a significant positive impact on daily activities. H2a1 refers to the positive impact of facility accessibility on walking, H2a2 refers to the positive impact of facility accessibility on outdoor exercise. H2b1 refers to the positive impact of mobility convenience on walking, H2b2 refers to the positive impact of mobility convenience on outdoor exercise. H2c1 refers to the positive impact of spatial environmental safety on walking, H2c2 refers to the positive impact of spatial environmental safety on outdoor exercise. H2d1 refers to the positive impact of facility layout safety on walking, H2d2 refers to the positive impact of facility layout safety on outdoor exercise. H2e1 refers to the positive impact of landscape environment comfort on walking, H2e2 refers to the positive impact of landscape environment comfort on outdoor exercise. H2f1 refers to the positive impact of positive impact of site spatial comfort on walking; H2f2 refers to the positive impact of site spatial comfort on outdoor exercise.

H3: Daily activities have a significant positive impact on the health of older adults. H3a1 refers to the positive impact of walking on physical health, H3a2 refers to the positive impact of walking on mental health. H3b1 refers to the positive impact of outdoor exercise on physical health, H3b2 refers to the positive impact of outdoor exercise on mental health.

### 2.2 Research sites

Fuzhou presents a compelling study area due to the pronounced conflict between high-density urban development and the urgent need for a healthy living environment for older adults within the community. The city’s unique geography, bordered by mountains on three sides and the sea on the fourth, creates a spatial configuration that simultaneously acts as a natural barrier and limits urban expansion. Fuzhou is currently undergoing a process of integrated development, resulting in constrained land availability for urban growth and a reduction in accessible living space for older adults. Moreover, in recent years, the Fuzhou municipal government has actively promoted the establishment of pedestrian-oriented urban systems and recreational spaces designed to support the health and mobility of older adults. Initiatives such as the creation of urban ecological trails, exemplified by the Jinniu Mountain Fudao, are not only supported by the government but also foster an environment conducive to community health and wellness.

**Figure 2:**
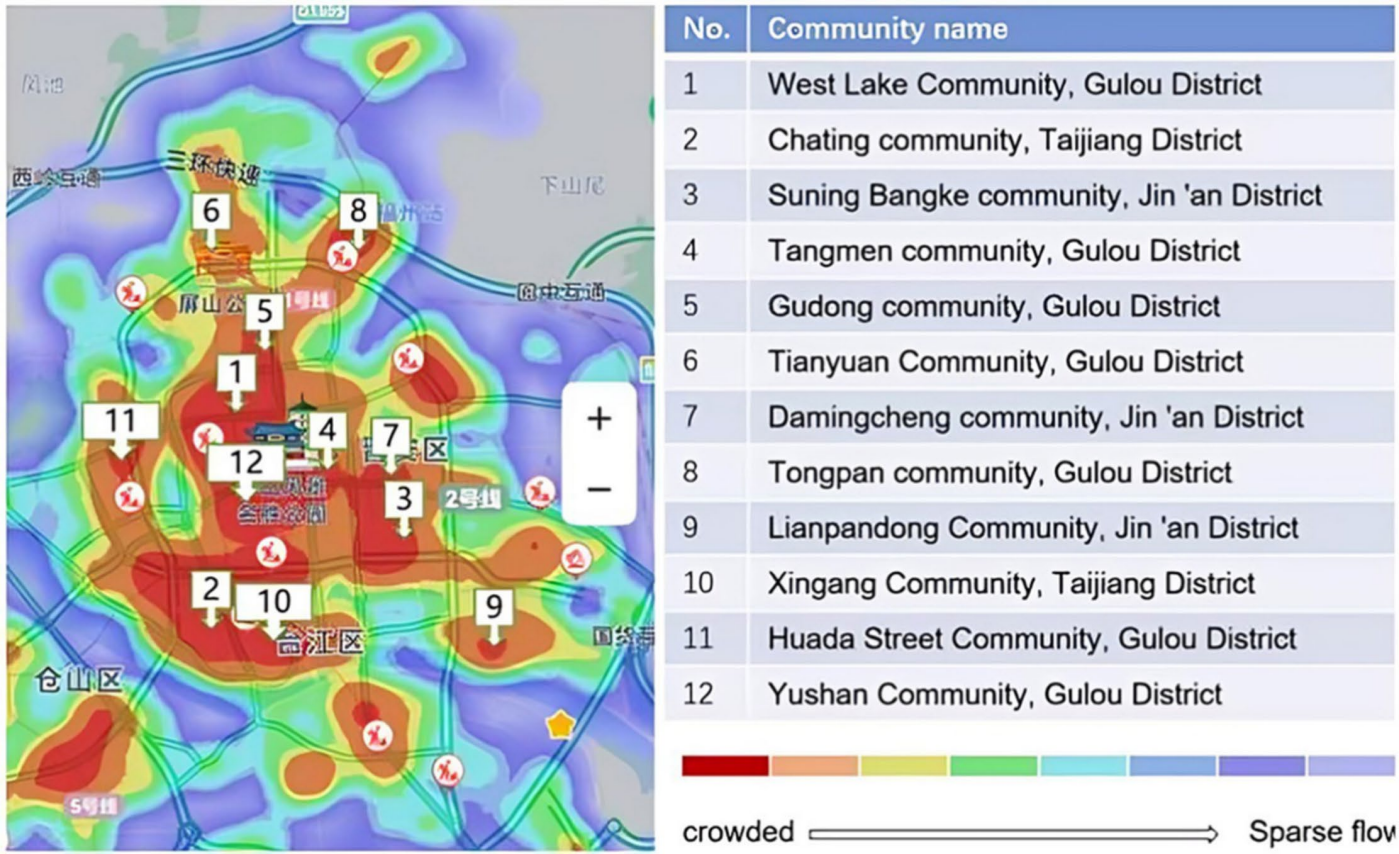
Heat map of sample neighborhoods.

Consequently, three urban districts in Fuzhou City—Gulou District, Taijiang District, and Jinan District—were selected as the study area. When selecting sample communities, communities that met the following three criteria were identified based on the evidence—based principles of gerontology and urban planning studies: a senior population exceeding 15% (40), the presence of a service station within the community (41), and at least one park located within 500 meters of the community (GB 50180–2018) (42, 43). This selection was informed by various factors, including the community support provided by the community council. Utilizing Baidu heat maps and on-site validation, twelve neighborhoods with high pedestrian traffic were identified as sample neighborhoods (Figure 2).

### 2.3 Data collection

The data obtained in this study were combined with data from preliminary research conducted for a settlement planning course in the urban and rural planning program at the College of Architecture and Urban Planning, Fujian University of Technology. The participants in the questionnaire survey were second-year undergraduate students (*n* = 24) of urban and rural planning. The trainers were members of the Fujian Provincial Natural Science Foundation (grant number 2022J05192) and teachers of the settlement planning course in the College of Architecture and Urban Planning, Fujian University of Technology. The trainers provided formal training to the data collectors consisting of three parts (Table 2). Twenty-four trained data collectors were divided into 12 groups to administer the questionnaire to residents of the 12 sample communities.

**Table 2:** Training steps.

Training steps	Training contents
Step1	Detailed explanations of each questionnaire question, including examples and photographs, as well as in-depth explanations of uncommon questions that are still difficult to answer
Step2	Data collection was pre-researched in sample communities to screen for difficult questions
Step3	Comparative analysis of difficult questions, harmonization of answers and improved accuracy of data collection

The data for this study were categorized into three main areas: the community built environment, daily activities, and the health of older adults. Data collection involved a combination of online and offline questionnaires. A preliminary version of the questionnaire was developed, followed by a pilot study conducted in May 2022. A total of 120 questionnaires were distributed, with ten allocated to each sampled community. Of the distributed questionnaires, fifteen were returned, and 105 were deemed valid. Following the pilot study’s findings, adjustments were made to the questionnaire to include inquiries related to the community built environment, daily activities, and the health of older adults.

The formal questionnaire commenced on September 17, 2022, and was disseminated in the 12 selected communities using a combination of online and offline approaches. The community sampling survey was completed through the steps of preliminary preparation, pilot survey, formal investigation, and data processing (Table 3).

**Table 3:** Community sampling survey steps.

Research phase	Detailed procedures	Timeline
Preliminary preparation	1. Identified communities with older adult population exceeding 15% using demographic data 2. Filtered target areas through spatial analysis of community service centers and parks within 500-meter coverage 3. Utilized Baidu Heatmap (a crowd density visualization tool) to identify high-traffic zones	April 2022
Pilot survey	1. Distributed 10 questionnaires in each of 12 sampled communities 2. Refined questionnaire content and phrasing based on feedback 3. Finalized sample size allocation strategy for formal survey	May 2022
Formal investigation	1. Deployed 24 trained investigators in 12 teams for field surveys 2. Implemented hybrid data collection (online/offline questionnaires) 3. Conducted weekly data consolidation and problem resolution	March–June 2023
Data processing	1. Eliminated invalid responses and verified data completeness 2. Established raw database architecture 3. Executed systematic data cleaning and transformation procedures	September–December 2023

Older adults in the community were recruited to participate in the study by offering a carton of eggs valued at approximately 6 RMB as an incentive for completing the questionnaire. Upon questionnaire completion, participants received the voucher promptly. Inclusion criteria for participation were: (1) age 60 years or older; (2) permanent residence in the selected communities for at least one year; (3) ability to understand and respond to the questionnaire either independently or with assistance; and (4) willingness to provide informed consent. Exclusion criteria were: (1) severe cognitive impairment preventing reliable response to questionnaires; (2) acute illness or hospitalization during the survey period; (3) inability to communicate effectively; and (4) temporary residents or those who had lived in the community for less than one year (44–46).

The online survey was administered using the questionnaire tool Star^1^. This platform provided a user-friendly web interface that elaborated on each question option. The offline survey took place in high-traffic areas within the community. Data collectors were tasked with explaining the question options either online or in person. They utilized photo examples to aid older adults in comprehending the questions and accurately completing the questionnaire. In the 12 sample communities, we distributed 595 questionnaires, including 432 online and 163 offline. We collected 494 valid questionnaires, including 356 online and 138 offline (Table 4). The questionnaire recovery validity rate stood at 84.04%, with online recoveries at 82.40% and offline at 84.66%.

**Table 4:** Sample distribution in each sample community.

Community name	Number of online questionnaires	Number of offline questionnaires	Total number of valid questionnaires
West Lake Community	39	12	51
Chating Community	35	10	45
Suning Bangke community	42	14	56
Tangmen Community	33	12	45
Gudong Community	38	11	49
Tianyuan Community	34	12	46
Damingcheng community	31	12	43
Tongan Community	29	11	40
Lianpandong Community	25	11	36
Xingang Community	22	10	32
Huada Street Community	15	10	25
Yushan Community	13	13	26
Total	356	138	494

From the perspectives of age, personal economic condition, physical condition and education (47), the statistics are as follows (Table 5).

**Table 5:** Socio-economic characteristics of the older adults (*n* = 494).

Socio-economic characteristics	Specific classification	Number	Percentage (%)
Gender	Female	282	57.0
	Male	212	43.0
Age	60–74 years (young-old)	283	57.3
	75–89 years (old-old)	203	41.1
	≥90 years (long-lived older adult)	8	1.6
Educational level	Primary school or below	266	53.8
	Junior high school	138	27.9
	Senior high school or vocational school	57	11.5
	College or above	33	6.7
Health status	Self-care	389	78.7
	Assisted living	98	19.8
	Nursing care	7	1.4
Monthly income level (CNY)	0–3,000	322	65.1
	3,000–6,000	81	16.4
	6,000–9,000	19	3.8
	≥9,000	19	3.8
	Unwilling to disclose	53	10.7

#### 2.3.1 Community built environmental data collection

For facility accessibility, the existing literature underscores the importance of accessibility to various facilities—such as cultural, sports, educational, healthcare, and commercial services—for older adults. For mobility convenience, the literature has examined the impact of pedestrian network connectivity, pedestrian crossing accessibility, and public transit accessibility on the well-being of older adults. The accessibility of entrances and exits is important for older adult residents traveling long distances, and this paper introduces the accessibility of entrances and exits into the study. Regarding the safety of the spatial environment, the literature has examined the safety of transportation environment, pavement quality, and the impact of noise on older adults. Concerning facility layout safety, studies have investigated the effects of barrier-free facilities and security monitoring. Interviews revealed that older adults often have the habit of walking after meals and express concerns about sudden health issues; thus, attention must be given to the coverage of nighttime lighting and the accessibility of emergency rescue systems. For the comfort of the landscape environment, the literature identifies several factors, including the ease of landscape vignettes, visual richness, corridor smoothness, green visibility, and tree shading rates. In terms of site spatial comfort, existing research has focused on the impacts of street cleanliness and the convenience of walking rest facilities, as well as the availability of rain and shade structures. Interviews indicated that a diverse range of functions along the street façade attracts older adults to go out, prompting this paper to introduce research on the functional richness of street facades. The community built environment data collection was based on six main dimensions (Table 6). Respondents were asked to evaluate the strengths and weaknesses of each factor on a five-point scale (1 = very poor, 2 = poor, 3 = normal, 4 = good, 5 = very good).

**Table 6:** Community built environment data collection.

Community built environment	Contributing factor
Facility accessibility	Accessibility to cultural facilities, accessibility to sports facilities, accessibility to educational facilities, accessibility to healthcare facilities, accessibility to care facilities, accessibility to commercial services
Mobility convenience	Accessibility to entrances and exits, pedestrian network connectivity, public transit accessibility, pedestrian crossing accessibility
Spatial environment safety	Safety of the transportation environment, pavement quality, noise management
Facility layout safety	Completeness of barrier-free facilities, coverage rate of nighttime lighting facilities, comprehensiveness of security monitoring facilities, accessibility of emergency response systems
Landscape environment comfort	Aesthetic and recreational appeal of landscape features, visual richness of landscape design, unobstructed clarity of landscape visual corridors, proportion of greenery in the visual field, tree canopy shading ratio
Site spatial comfort	Cleanliness of streets, functional diversity of street-facing facades, accessibility of walking rest facilities, convenience of rain and sun protection facilities

#### 2.3.2 Daily activity data collection

Based on the aforementioned literature review, daily activities included walking and outdoor exercise, categorized by types of activities, daily frequency, and duration of each session (Table 7). Respondents were asked to select the duration of their participation (1 = 0–5 min per day, 2 = 5–15 min per day, 3 = 15–30 min per day, 4 = 30–60 min per day, 5 = 60 min per day and above).

**Table 7:** Daily activities data collection.

Daily activities	Contributing factor
Walking	Daily walking frequency, duration of each walking session, types of walking activities
Outdoor exercise	Daily outdoor exercise frequency, duration of each outdoor exercise session, types of outdoor exercise activities

#### 2.3.3 Health of older adults data collection

According to the literature review, ease of mobility, quality of sleep, dietary habits, and chronic health conditions are associated with physical health (48–51), while cognitive health, emotional health, happiness and satisfaction, and psychological resilience are related to mental health (52–55) (Table 8). Consequently, the relevant factors pertaining to the health of the older adult have been summarized. Participants were requested to evaluate their physical condition (Table 9).

**Table 8:** Contributing factors to health of older adults and supporting research.

Health of older adults	Contributing factor	Research support and description	Related research literature
Physical health	Ease of mobility	Mobility is a key determinant of healthy aging in older adults, and its decline predicts increasing functional impairment and dependency.	Ferrucci et al. (48)
	Quality of sleep	Sleep plays a vital role in brain function and systemic physiology across many body systems.	Goran et al. (49)
	Dietary habits	Dietary habit assessment is significantly valuable in predicting the maintenance of physical function in older adults.	Xing et al. (50)
	Chronic health conditions	Chronic diseases strongly affect the daily functioning of the older adult and is a powerful predictor of their health status. Each additional chronic disease increases the risk of functional decline by 37%.	Mao et al. (51)
Mental health	Cognitive health	Cognitive health is strongly associated with functional changes. Better cognitive function is linked to higher quality of life in older adults.	Pan et al. (52)
	Emotional health	Assessment of emotional health is crucial for the mental health of the older adult and holds significant public health importance.	Lopez et al. (53)
	Happiness and satisfaction	Higher life satisfaction is associated with better health outcomes and longer lifespan.	Padmanabhanunni et al. (54)
	Psychological resilience	Assessment of psychological resilience is crucial for understanding the capacity of older adults to cope with health challenges and is an important indicator of their mental health resources.	Macleod et al. (55)

**Table 9:** Questionnaire items and scoring scales for health of older adults assessment.

Health of older adults	Contributing factor	Sample questionnaire item	Scoring scale
Physical health	Ease of mobility	“How would you rate your ability to walk independently?”	1 = Extremely difficult, 2 = Difficult, 3 = Moderate, 4 = Easy, 5 = Extremely easy
	Quality of sleep	“How would you evaluate your overall sleep quality during the past month?”	1 = Very poor, 2 = Poor, 3 = Adequate, 4 = Good, 5 = Excellent
	Dietary habits	“How frequently do you consume fresh vegetables and fruits?”	1 = Never, 2 = Rarely, 3 = Occasionally, 4 = Frequently, 5 = Daily
	Chronic health conditions	“To what extent do chronic conditions impact your daily functioning?”	1 = Severe impact, 2 = Moderate impact, 3 = Mild impact, 4 = Minimal impact, 5 = No impact
Mental health	Cognitive health	“Compared to one year ago, how would you rate your memory capacity?”	1 = Significantly declined, 2 = Slightly declined, 3 = Unchanged, 4 = Good, 5 = Excellent
	Emotional health	“During the past two weeks, how often have you experienced feelings of depression?”	1 = Nearly always, 2 = Frequently, 3 = Sometimes, 4 = Rarely, 5 = Never
	Happiness and satisfaction	“Overall, you are satisfied with your life”	1 = Strongly disagree, 2 = Disagree, 3 = Neutral, 4 = Agree, 5 = Strongly agree
	Psychological resilience	“When confronted with difficulties, you can identify solutions”	1 = Not at all true, 2 = Rarely true, 3 = Sometimes true, 4 = Often true, 5 = True nearly all the time

#### 2.3.4 Data analysis

Likert scales were employed to evaluate the data. Latent variables, including facility accessibility, mobility convenience, spatial environment safety, facility layout safety, landscape environment comfort, site spatial comfort, walking, outdoor exercise, and both physical and mental health, were considered, with their corresponding factors treated as observed variables (Table 10). These variables were inputted into SPSS 27.0 and AMOS 27.0 statistical software for structural equation modeling analysis.

**Table 10:** Latent and observed variables.

Latent variable	Observed variable (y)
Facility accessibility (F1)	Accessibility to cultural facilities (A1), accessibility to sports facilities (A2), accessibility to education facilities (A3), accessibility to healthcare facilities (A4), accessibility to care facilities (A5), accessibility to commercial services (A6)
Mobility convenience (F2)	Accessibility to entrances and exits (A7), pedestrian network connectivity (A8), public transit accessibility (A9), pedestrian crossing accessibility (A10)
Spatial environment safety (F3)	Safety of the transportation environment (A11), pavement quality (A12), noise management (A13)
Facility layout safety (F4)	Completeness of barrier-free facilities (A14), coverage rate of nighttime lighting facilities (A15), comprehensiveness of security monitoring facilities (A16), accessibility of emergency response systems (A17)
Landscape environment comfort (F5)	Aesthetic and recreational appeal of landscape features (A18), visual richness of landscape design (A19), unobstructed clarity of landscape visual corridors (A20), proportion of greenery in the visual field (A21), tree canopy shading ratio (A22)
Site spatial comfort (F6)	Cleanliness of streets (A23), functional diversity of street-facing facades (A24), accessibility of walking rest facilities (A25), convenience of rain and sun protection facilities (A26)
Walking (F7)	Daily walking frequency (B1), duration of each walking session (B2), types of walking activities (B3)
Outdoor exercise (F8)	Daily outdoor exercise frequency (B4), duration of each outdoor exercise session (B5), types of outdoor exercise activities (B6)
Physical health (F9)	Ease of mobility (C1), quality of sleep (C2), dietary habits (C3), chronic health conditions (C4)
Mental health (F10)	Cognitive health (C5), emotional health (C6), happiness and satisfaction (C7), psychological resilience (C8)

Data analysis for reliability and validity was conducted using SPSS version 27.0. The study focused on assessing the reliability and validity of the grade level variables in the questionnaire. Reliability testing involved Cronbach’s alpha analysis and modified item-total correlation (CITC) analysis.

The results indicated a Cronbach’s alpha coefficient of 0.904 for the overall data, with coefficients above 0.8 for both latent and observed variables (56). The item correlation coefficients all exceeded 0.50, indicating strong relationships between variables and confirming good reliability according to established guidelines (57). Validity assessment utilized the KMO (Kaiser-Meyer-Olkin) test and Bartlett’s test of sphericity. The KMO value was 0.910, well above the 0.80 threshold considered “meritorious” for factor analysis (58). Additionally, Bartlett’s test of sphericity was statistically significant (*p* ≤ 0.001) (59).

Model testing and analysis were conducted subsequently. The study data underwent analysis utilizing AMOS software, and model testing was executed employing the maximum likelihood estimation (MLE) method for the estimation of model parameters. The chi-square degrees of freedom ratio (*χ*/df), goodness-of-fit index (GFI), root mean square error of approximation (RMSEA), comparative fit index (CFI), normal fit index (NFI), and adjusted GFI (AGFI) were utilized to assess the model fit (Table 11). The findings indicated that all the indices satisfied the standard range criteria, signifying a good fit and an ideal model.

**Table 11:** Fitting index of the model.

Universal index	(An official) standard	Parameter value
x2/df	< 3	2.6
GFI	> 0.900	0.835
RMSEA	< 0.050	0.044
CFI	> 0.950	0.948
NFI	> 0.900	0.864
AGFI	> 0.900	0.811

Standardized path coefficients were examined for both observed and latent variables within the community health support environment. The results were statistically significant (*p* ≤ 0.05). All primary paths were statistically significant.

## 3 Results

**Figure 3:**
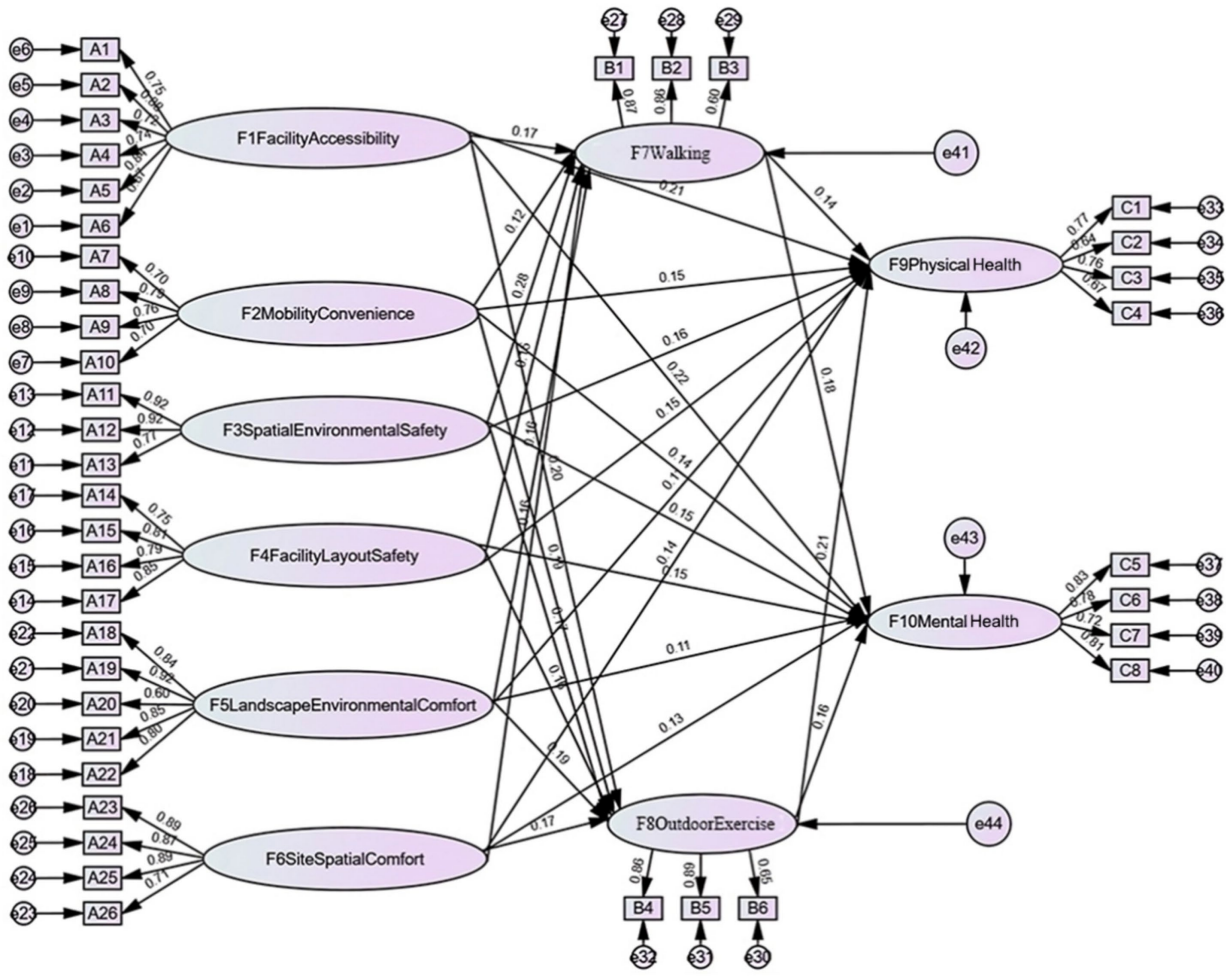
Pathways of influence of community built environment, daily activities and health of older adults.

The findings from the structural equation modeling analysis indicated that all proposed hypothetical models were accepted (Figure 3).

### 3.1 Direct effects of the community built environment on the health of older adults

The findings indicate that the research hypothesis models H1a1, H1a2, H1b1, H1b2, H1c1, H1c2, H1d1, H1d2, H1e1, H1e2, H1f1, and H1f2 (Figures 1, 3) were supported. The community built environment was shown to have a significant direct impact on the physical and mental health of older adults (Table 12). Among these factors, facility accessibility exerted the greatest combined impact on older adult health (effect coefficient: 0.43). This encompassed direct effects on physical health (impact coefficient: 0.21) and mental health (impact coefficient: 0.22). High-quality facility accessibility enables older adults to participate more conveniently in cultural and sports activities and healthcare services, thereby effectively delaying chronic diseases and enhancing well-being. The safety of spatial environment and the safety of facility layout, also significantly affect the health of older adults (combined impact coefficients of 0.31 and 0.30, respectively). Safety reduces the risk of falls and transportation accidents, providing an important safeguard for the physical and mental health of the older adults.

**Table 12:** Coefficient of direct effect of community built environment on health of older adults.

Impact factors	Physical health (F9)	Mental health (F10)	Health of older adults
Facility accessibility (F1)	0.21	0.22	0.43
Mobility convenience (F2)	0.15	0.14	0.29
Spatial environmental safety (F3)	0.16	0.15	0.31
Facility layout safety (F4)	0.15	0.15	0.30
Landscape environment comfort (F5)	0.11	0.11	0.22
Site spatial comfort (F6)	0.14	0.13	0.27

### 3.2 Effects of the community built environment on the daily activities of older adults

The hypothetical models H2a1, H2a2, H2b1, H2b2, H2c1, H2c2, H2d1, H2d2, H2e1, H2e2, H2f1, and H2f2 were validated (Figures 1, 3). Key factors of the built environment—including facility accessibility, mobility convenience, spatial environmental safety, facility layout safety, landscape environmental comfort, and site spatial comfort—were found to have significant positive effects on walking and outdoor exercise (Table 13). Among these factors, spatial environmental safety had the most pronounced impact on daily activities (impact coefficient: 0.45), Walking was particularly affected by the safety of the spatial environment (impact coefficient of 0.28). Facility accessibility had the most significant impact on outdoor activities (impact coefficient of 0.20). This result suggests that optimizing the built environment of a community can significantly improve the quality of daily activities for older adults.

**Table 13:** The influence coefficient of community built environment on daily activities.

Influence coefficient	Walking (F7)	Outdoor exercise (F8)	Daily activities
Facility accessibility (F1)	0.17	0.20	0.37
Mobility convenience (F2)	0.12	0.19	0.31
Spatial environmental safety (F3)	0.28	0.17	0.45
Facility layout safety (F4)	0.16	0.16	0.32
Landscape environment comfort (F5)	0.16	0.19	0.35
Site spatial comfort (F6)	0.16	0.17	0.33

### 3.3 Impact of daily activities on the health of older adults

Hypothesized models H3a1, H3a2, H3b1 and H3b2 were supported (Figures 1, 3). Daily activities played a crucial mediating role between the community built environment and the health of older adults (Table 14). Walking was particularly effective in enhancing mental health (impact coefficient of 0.18), and outdoor exercise emerged as the strongest contributor to physical health (impact coefficient of 0.21). These findings suggest that both physical and mental health can be significantly improved by enhancing the community built environment to promote daily activities among older adults.

**Table 14:** The influence coefficient of daily activities on health of older adults.

Influence coefficient	Physical health (F9)	Mental health (F10)	Health of older adults
Walking (F7)	0.14	0.18	0.32
Outdoor exercise (F8)	0.21	0.16	0.37

Among the three observed variables of walking the frequency of daily walking and the duration of each walking had the most significant impact on the health of older adults (Table 15). In community planning, it is essential to arrange suitable activity sites and prioritize the construction of community walking paths to enhance the walking environment. Among the additional three activity variables observed, daily outdoor exercise frequency and daily outdoor exercise time had the greatest influence on the health of older adults (Table 16). Community planning should focus on improving exercise areas and equipment to the fullest extent possible.

**Table 15:** Factors influencing walking.

Variables	Factors
Daily walking frequency	0.87
Walking time per session	0.86
Types of walking	0.6

**Table 16:** Factors influencing outdoor exercise.

Variables	Factors
Daily outdoor exercise frequency	0.86
Outdoor exercise time per session	0.89
Types of outdoor sports	0.65

### 3.4 Indirect effects of the community built environment on the health of older adults

The community built environment indirectly influences the health of older adults through daily activities (Table 17). These daily activities play a crucial mediating role in the relationship between the community built environment and the health of older adults. Facility accessibility indirectly affects physical health (0.0658 [0.17*0.14 + 0.20*0.21]) and mental health (0.0626 [0.17*0.18 + 0.20*0.16]) through walking and outdoor exercise. Mobility convenience indirectly affects physical health (0.0567 [0.12*0.14 + 0.19*0.21]) and mental health (0.052 [0.12*0.18 + 0.19*0.16]) through walking and outdoor exercise. Spatial environment safety indirectly affects physical health (0.0749 [0.28*0.14 + 0.17*0.21]) and mental health (0.0776 [0.28*0.18 + 0.17*0.16]) through walking and outdoor exercise. Facility layout safety indirectly affects physical health (0.056 [0.16*0.14 + 0.16*0.21]) and mental health (0.0544 [0.16*0.18 + 0.16*0.16]) through walking and outdoor exercise. Landscape environmental comfort indirectly affects physical health (0.0623 [0.16*0.14 + 0.19*0.21]) and mental health (0.0592 [0.16*0.18 + 0.19*0.16]) through walking and outdoor exercise. Site spatial comfort indirectly affects physical health (0.0581 [0.16*0.14 + 0.17*0.21]) and mental health (0.056 [0.16*0.18 + 0.17*0.16]) through walking and outdoor exercise. Overall, spatial environmental safety had the strongest mediating effect in indirectly promoting the health of older residents.

**Table 17:** Coefficient of indirect effect of community built environment on health of older adults.

Impact factors	Physical health	Mental health	Health of older adults
Facility accessibility (F1)	0.066	0.063	0.128
Mobility convenience (F2)	0.057	0.052	0.109
Spatial environmental safety (F3)	0.075	0.078	0.153
Facility layout safety (F4)	0.056	0.054	0.110
Landscape environment comfort (F5)	0.062	0.059	0.122
Site spatial comfort (F6)	0.058	0.056	0.114

## 4 Discussion

### 4.1 Comprehensive effects of the community built environment on the health of older adults

The relationship between the community built environment and the health of older adults can be interpreted through a social-ecological theory, which posits that health outcomes emerge from dynamic interactions across multiple levels, including individual behaviors, community resources, and broader environmental contexts (60, 61). Our findings align with this framework, revealing that the built environment serves as a critical meso-level system that both directly shapes health and indirectly facilitates health-promoting behaviors through daily activities. These direct and indirect impacts together constitute the overall influence of the community built environment on the health of older adults (Table 18).

**Table 18:** Coefficient of influence of the community built environment on the health of older adults.

Impact factors	Direct influence coefficient of physical health	Indirect influence coefficient of physical health	Comprehensive influence coefficient of physical health	Direct influence coefficient of mental health	Indirect influence coefficient of mental health	Comprehensive influence coefficient of mental health
Facility accessibility (F1)	0.210	0.066	0.276	0.220	0.063	0.283
Mobility convenience (F2)	0.150	0.057	0.207	0.140	0.052	0.192
Spatial environmental safety (F3)	0.160	0.075	0.235	0.150	0.078	0.228
Facility layout safety (F4)	0.150	0.056	0.206	0.150	0.054	0.204
Landscape environment comfort (F5)	0.110	0.062	0.172	0.110	0.059	0.169
Site spatial comfort (F6)	0.140	0.058	0.198	0.130	0.056	0.186

The combined impact coefficient of facility accessibility on the health of older adults is 0.5584, comprising a coefficient of 0.2758 for physical health and 0.2826 for mental health. This indicates that a one-unit improvement in facility accessibility corresponds to a 0.5584-unit enhancement in the overall health of older adults. The combined impact coefficient of mobility convenience on the health of older adults is 0.3987, comprising a coefficient of 0.2067 for physical health and 0.192 for mental health. This indicates that a one-unit improvement in mobility convenience results in a 0.3987-unit enhancement in the health of older adults. The combined impact coefficient of spatial environment safety on the health of older adults is 0.4625, comprising coefficients of 0.2349 for physical health and 0.2276 for mental health. This indicates that a one-unit improvement in spatial environment safety leads to a 0.4625-unit enhancement in the health of older adults. The combined impact coefficient of facility layout safety on the health of older adults is 0.4104, with a coefficient of 0.206 for physical health and 0.2044 for mental health. This indicates that a one-unit increase in facility layout safety contributes to a 0.4104-unit enhancement in the health of older adults. The combined impact coefficient of landscape environmental comfort on the health of older adults is 0.3415, comprising coefficients of 0.1723 for physical health and 0.1692 for mental health. This indicates that a one-unit improvement in landscape environmental comfort corresponds to a 0.3415-unit enhancement in the health of older adults. The combined impact coefficient of site spatial comfort on the health of older adults is 0.3841, with coefficients of 0.1981 for physical health and 0.186 for mental health. This indicates that a one-unit improvement in site spatial comfort results in a 0.3841-unit enhancement in the health of older adults (Table 19).

**Table 19:** Combined impact coefficient of community built environment on the health of older adults.

Impact factors	Physical health	Mental health	Health of older adults
Facility accessibility (F1)	0.276	0.283	0.558
Mobility convenience (F2)	0.207	0.192	0.399
Spatial environmental safety (F3)	0.235	0.228	0.463
Facility layout safety (F4)	0.206	0.204	0.410
Landscape environment comfort (F5)	0.172	0.169	0.342
Site spatial comfort (F6)	0.198	0.186	0.384

The findings of the study clearly indicate that the community built environment plays a crucial role in maintaining and enhancing the health of older adults. Facility accessibility is particularly significant for the physical and mental health of older adults and should be a primary focus in community built environment planning. This aligns with previous research emphasizing the role of environmental affordances as a driving force for agency in older adults (62). Emphasis should be placed on optimizing the distribution and functional design of these facilities (63).

### 4.2 Practical recommendations for optimizing the built environment of communities to promote the health of older adults

In terms of facility accessibility, prioritizing the equitable distribution of amenities is critical to meet the diverse needs of older residents in every building within the community. Establishing centralized older adult activity centers in core areas can function as key hubs, promoting social interaction and enhancing participation in activities.

Regarding mobility convenience, implementing community shuttle services to connect key residential areas with community entrances and exits can significantly improve mobility. Enhancing the internal pedestrian network will boost connectivity, while optimizing bus routes and constructing pedestrian bridges or gently sloped ramps at major crossings can ensure safer and more convenient street crossings for older residents.

For spatial environment safety, measures should prioritize the safety of transportation systems. Installing dividers to separate pedestrian and vehicular traffic, ensuring smooth road surfaces, and maintaining obstacle-free walking paths and activity sites are essential. Additionally, installing noise monitoring devices can help manage and reduce noise pollution effectively.

With respect to the safety of facility layout, improving barrier-free facilities is imperative. Introducing night-time induction lighting systems along walking paths and activity areas can enhance the safety of evening activities. High-definition surveillance systems should be installed in critical locations, such as entrances and activity plazas, to strengthen security. Furthermore, first aid stations or emergency alarm systems should be established in activity venues and along main access routes to ensure rapid response in emergencies.

In terms of landscape environmental comfort, increasing green spaces and flowerbeds can enhance the visual appeal of walking corridors. Small-scale natural interaction points, such as water features or fountains, can create relaxing environments, while designing themed landscape areas can offer diverse recreational opportunities tailored to the preferences of older residents.

For site spatial comfort, installing awnings and pavilions along major roads and rest areas can provide shade and shelter. Benches placed at 200-meter intervals in walking corridors, fitness areas, and public plazas can ensure sufficient resting spaces for older residents during their activities. Additionally, regular cleaning and maintenance of streets and pathways should be undertaken to keep them clear of clutter, prevent haphazard parking, and enhance the overall walking experience.

Particular emphasis should be placed on designing accessible facilities. Increasing the number of small fitness areas and outdoor activity spaces, especially along major walking corridors, ensures equitable access for older adults across different residential zones. The density of community clinics and health education points should also be increased to provide health counseling and basic diagnostic services. Facilities such as daycare centers and community canteens should be conveniently located within community centers and designed with barrier-free access. Small commercial service points, including supermarkets and food markets, should be rationally distributed to ensure accessibility within a 5–10 min walk (Table 20).

**Table 20:** Factors influencing facility accessibility.

Variables	Factors
Accessibility to commercial services	0.87
Accessibility of care facilities	0.84
Accessibility of health-care facilities	0.74
Accessibility of educational facilities	0.72
Accessibility of sports facilities	0.88
Accessibility of cultural facilities	0.75

### 4.3 Key role of daily activities in health promotion

The mediating effect of daily activities highlights the indirect influence of community design on health outcomes. Outdoor exercise and walking are not only essential forms of community engagement for older adults but also crucial contributors to their overall health. Walking, in particular, has been shown to significantly enhance mental health, while outdoor exercise is particularly effective in improving physical function. These findings indicate that creating suitable activity spaces and pedestrian networks can greatly enhance the health of older adults.

### 4.4 Research limitations and extensions

#### 4.4.1 Updating methods of data acquisition and means of analysis

This study collected data through questionnaires; however, this method has limitations, primarily due to the small sample size and the uncertainty associated with relying on a single data source. Future research should implement a multi-channel data collection and integration strategy to enhance the comprehensiveness and accuracy of the data. Specific recommendations include: first, fostering cross-sectoral collaboration with public transportation departments, community hospitals, and social networking platforms to gather data related to the community environment, daily activities, and health status. Second, diversifying data integration by utilizing remote sensing technology, geo-tagged time-series data, street view images, and residents’ activity trajectories, in conjunction with hospital patient information and personal health reports, to provide a comprehensive overview of the health behavior characteristics of older community residents. Finally, innovative analytical tools should be employed to examine the correlations among multi-source data by adopting cross-modal data integration technology, thereby enabling a thorough exploration of the comprehensive impact of the community built environment on the health of the older adults. This approach aims to provide a high-precision foundation for policy formulation.

#### 4.4.2 Addressing the needs of residents across different age groups

Current research primarily focuses on the older adult, often neglecting the health needs of residents from various age groups within the community concerning the built environment. To develop a more inclusive model that addresses the health implications of the built environment, the following considerations should be taken into account: First, broaden the scope of research to comprehensively analyze the diverse needs of young, middle-aged, and older adult residents in community health services, while also exploring their interactions with the environment. Second, optimize the functional layout of the community and dynamically adjust the configuration of public facilities based on the needs of residents across multiple age groups, ensuring that all individuals can benefit. Finally, establish an intergenerational co-construction model that encourages the active participation of residents from different age groups in shaping the community built environment through intergenerational interaction programs, thereby enhancing the overall health of the community.

#### 4.4.3 Conducting multi-regional comparative studies

Since the regional sample of this study is limited to Fuzhou City, it is challenging to generalize the findings to other cities or regions. Therefore, future research should focus on the following: First, expanding the scope of the study to include similar investigations in various cities, regions, and countries to examine the impact of geographic differences on the health needs of older adult residents in the community. Second, conducting a differentiation analysis to facilitate in-depth comparisons of cultural backgrounds, economic conditions, and community planning across different regions, thereby refining replicable and scalable strategies for building healthy communities. Finally, establishing a data-sharing platform will promote the accessibility and sharing of community health research data through international and inter-regional collaboration, supporting cross-regional comparative research.

## 5 Conclusion

A meticulously designed community environment is instrumental in promoting the physical and mental well-being of older adults. Such an environment can significantly enhance their levels of physical activity, mitigate mental stress, decrease fatigue, and cultivate a sense of belonging within the community. This study seeks to investigate the daily routines of older adult individuals to elucidate the relationships among the community environment, daily activities, and the health of older adults, as well as the mechanisms through which these factors interact.

Research indicates that the physical infrastructure of a community can significantly influence the well-being of older adults. Firstly, the built environment can directly enhance the health of older adults, with accessibility identified as the most critical factor. Secondly, the built environment can indirectly affect the health of older adults by facilitating their daily activities, in which spatial environmental safety plays a pivotal role. Thirdly, the daily routines of older adults act as a mediator in the relationship between the built environment and their health. This mediation is particularly pronounced when the physical environment impacts the health of older adults who participate in outdoor exercise. Finally, the accessibility of facilities has the most substantial cumulative effect on the health of older adults.

It is imperative to prioritize the strategic placement of diverse facilities within the framework of community planning and development to encourage older individuals to utilize and engage with these resources, thereby enhancing their overall well-being. Additionally, community recreational areas and pathways should be meticulously designed to motivate older adults to engage in regular physical activities. The referenced study offers a coherent framework for improving the health of older populations and proposes practical initiatives and strategies for the creation of a health-promoting environment.

## Data Availability

The original contributions presented in the study are included in the article/Supplementary material, further inquiries can be directed to the corresponding author.

## Footnotes

^1^ www.wjx.cn

## References

[1] Tine B Chris P. Can global cities be 'Age-friendly Cities'? Urban development and ageing populations. Cities. (2016) 55:94–100. doi: 10.1016/j.cities.2016.03.016, PMID: 40094570

[2] Poganik JR Gladyshev VN. We need to shift the focus of aging research to aging itself. Proc Natl Acad Sci USA. (2023) 120:e2307449120. doi: 10.1073/pnas.2307449120, PMID: 37682890 PMC10500180

[3] Liljas AEM Walters K Jovicic A Iliffe S Manthorpe J Goodman C. Strategies to improve engagement of 'Hard to Reach' older people in research on health promotion: a systematic review. BMC Public Health. (2017) 17:349–61. doi: 10.1186/s12889-017-4241-8, PMID: 28431552 PMC5399821

[4] Song Y Wang Y Zhou M Suo Z Wang X Li C. Association between the perceived built environment and health behaviors in older adults: a cross-sectional study from Beijing, China. BMC Geriatrics. (2024) 24:1–9. doi: 10.1186/s12877-024-05285-7, PMID: 39160474 PMC11331743

[5] Organisation WH. Constitution of World Health Organisation. Geneva: World Health Organisation. (1948). Available online at: https://www.who.int/about/governance/constitution

[6] Xu Y Pan C Yu H Zhan B. Correlation analysis of the Urban Community environment and health promotion among adults aged ≥ 55 years: the mediating role of physical activity. BMC Public Health. (2024) 24:2790. doi: 10.1186/s12889-024-20303-4, PMID: 39394113 PMC11470714

[7] Zheng Z Liu W Lu Y Sun N Chu Y Chen H. The influence mechanism of community-built environment on the health of older adults: from the perspective of low-income groups. BMC Geriatr. (2022) 22:590. doi: 10.1186/s12877-022-03278-y, PMID: 35842581 PMC9288733

[8] Vahabi S Lak A Panahi N. Driving the determinants of older People’s mental health in the context of urban resilience: a scoping review. BMC Geriatr. (2023) 23:711. doi: 10.1186/s12877-023-04387-y, PMID: 37919669 PMC10623797

[9] Lyu X Fan Y. The impact of home-and community-based services on the health of older adults: a Meta-analysis. SAGE Open. (2024) 14:787–95. doi: 10.1177/21582440241285674

[10] Bonaccorsi G Milani C Giorgetti D Setola N Naldi E Manzi F. Impact of built environment and neighborhood on promoting mental health, well-being, and social participation in older people: an umbrella review. Ann Ig. (2022) 35:213–39. doi: 10.7416/ai.2022.2534, PMID: 35788248

[11] Niculita-Hirzel H Hirzel AH Wild P. A Gis-based approach to assess the influence of the urban built environment on cardiac and respiratory outcomes in older adults. Build Environ. (2024) 253:111362. doi: 10.1016/j.buildenv.2024.111362

[12] Liu Y Guo Y Lu S Chan O Chui C Ho H. Understanding the long-term effects of public open space on older Adults' functional ability and mental health. Build Environ. (2023) 234:110126. doi: 10.1016/j.buildenv.2023.110126

[13] Xiao Y Chen S Miao S Yu Y. Exploring the mediating effect of physical activities on built environment and obesity for elderly people: evidence from Shanghai, China. Front Public Health. (2022) 10:853292. doi: 10.3389/fpubh.2022.853292, PMID: 35359789 PMC8961803

[14] Andersen AMJ Jervelund SS Maindal HT Hempler NF. Acquisition, application, and distribution of health literacy from culturally sensitive type 2 diabetes education among Arabic-speaking migrants in Denmark: a longitudinal qualitative analysis. Scand J Caring Sci. (2024) 38:523–35. doi: 10.1111/scs.13228, PMID: 38031875

[15] Zhao Z Zhehao Y Yihua M. Effect of the establishment of age-friendly communities on the life satisfaction levels of the elderly in Beijing. J Tsinghua Univ. (2024):1–10. doi: 10.16511/j.cnki.qhdxxb.2024.22.051

[16] Yafei Y Dongfeng Y Dan X Jinxin L. How built environment affects the mental health of urban older adults: contrasting perspective based on observation and perception. China City Plan Rev. (2022) 31:26–37. doi: 10.20113/j.ccpr.2022.03.003

[17] Barnett DW Barnett A Nathan A Cauwenberg JV Cerin E. Built environmental correlates of older Adults' Total physical activity and walking: a systematic review and Meta-analysis. The. Int J Behav Nutr Phys Act. (2017) 14:103–27. doi: 10.1186/s12966-017-0558-z, PMID: 28784183 PMC5547528

[18] Zhang Z Tang X Shen ZY Yang L. Built-environment determinants of active travel behavior of older adults in Xiamen, China. Int Rev Spat Plan Sust Dev. (2022) 10:130–45. doi: 10.14246/irspsd.10.4_130

[19] Shrivastava RB Shrivastava PS Joshi A. Use of technology to promote road safety: public health perspective. J Pharm Bioal Sci. (2024) 16:S2941–3. doi: 10.4103/jpbs.jpbs_1244_23, PMID: 39346226 PMC11426710

[20] Sallis JF Cerin E Kerr J Adams MA Owen N. Built environment, physical activity, and obesity: findings from the international physical activity and environment network (Ipen) adult study. Annu Rev Public Health. (2020) 41:119–39. doi: 10.1146/annurev-publhealth-040218-043657, PMID: 32237990

[21] Anrooij VV Koks-Leensen MCJ Cruijsen AVD Jansen H Velden KVD Leusink G. How can care settings for people with intellectual disabilities embed health promotion? J Appl Res Intell Dis. (2020) 33:1489–99. doi: 10.1111/jar.12776, PMID: 32627935 PMC7689850

[22] Wang L Kang J. Acoustic demands and influencing factors in facilities for the elderly. Appl Acoust. (2020) 170:107470. doi: 10.1016/j.apacoust.2020.107470

[23] Rosenberg DE Huang DL Simonovich SD Basia B. Outdoor built environment barriers and facilitators to activity among midlife and older adults with mobility disabilities. Gerontologist. (2013) 53:268–79. doi: 10.1093/geront/gns119, PMID: 23010096 PMC3605937

[24] Liu Q He H Yang J Feng X Lyu J. Changes in the global burden of depression from 1990 to 2017: findings from the global burden of disease study. J Psychiatr Res. (2019) 126:134–40. doi: 10.1016/j.jpsychires.2019.08.002, PMID: 31439359

[25] Shouyi W Xian L Hua F. Research on the prediction model of night lighting environment of college gymnasium based on visual comfort. Urban Arch. (2021) 12:29–31. doi: 10.19892/j.cnki.csjz.2021.07.06

[26] Padeiro M de Sao Jose J Amado C Sousa L Roma Oliveira C Esteves A. Neighborhood attributes and well-being among older adults in urban areas: a mixed-methods systematic review. Res Aging. (2022) 44:351–68. doi: 10.1177/0164027521999980, PMID: 33906556 PMC9039320

[27] Pan Z Liu Y Li Z. Can living in an age-friendly Neighbourhood protect older Adults' mental health against functional decline in China? Landsc Urban Plan. (2023) 240:104897. doi: 10.1016/j.landurbplan.2023.104897, PMID: 40094570

[28] Yaoqiong Z Zhenwei L. Optimization strategy of public space in old community based on health promotion of the elderly. Chin Landsc Architect. (2021) 37:56–61. doi: 10.19775/j.cla.2021.S2.0056

[29] Grey T Xidous D O'Neill D Collier M. Growing older urbanism: exploring the Nexus between ageing, the built environment, and urban ecosystems. Urban Transform. (2023) 5:1–13. doi: 10.1186/s42854-023-00053-z36694624

[30] Hassan A Deshun Z. Psychophysiological impact of touching landscape grass among older adults. J Urban Health. (2024) 101:792–803. doi: 10.1007/s11524-024-00875-7, PMID: 38739226 PMC11329456

[31] Leigh G Leigh A. Leafy localities, longer lives: a cross-sectional and spatial analysis. Landsc Urban Plan. (2024) 242:104947. doi: 10.1016/j.landurbplan.2023.104947

[32] Yang L Tang X Liu MJ. Using a system of equations to assess the determinants of the walking behavior of older adults. Transact GIS TG. (2022) 26:1339–54. doi: 10.1111/tgis.12916

[33] Booth FW Roberts CK Laye MJ. Lack of exercise is a major cause of chronic diseases. Compr Physiol. (2012) 2:1143. doi: 10.1002/cphy.c110025, PMID: 23798298 PMC4241367

[34] Wang L Li S Wei L Ren B Zhao M. The effects of exercise interventions on mental health in Chinese older adults. J Environ Public Health. (2022) 2022:2–11. doi: 10.1155/2022/7265718, PMID: 35844951 PMC9277469

[35] Parra-Rizo MA Díaz-Toro F Hadrya F Pavón-León P Cigarroa I. Association of co-Living and age on the type of sports practiced by older people. Sports. (2022) 10:200. doi: 10.3390/sports10120200, PMID: 36548497 PMC9785896

[36] Li L Li W Fan T. Baduanjin exercise improves cognitive function in older adults with mild cognitive impairment. J Nerv Ment Dis. (2024) 212:500–6. doi: 10.1097/NMD.00000000000001796, PMID: 39207292

[37] Voss MW Sutterer M Weng TB Burzynska AZ Fanning J Salerno E. Nutritional supplementation boosts aerobic exercise effects on functional brain systems. Am Physiol Soc. (2019) 126:77–87. doi: 10.1152/JAPPLPHYSIOL.00917.2017, PMID: 30382806 PMC6383642

[38] Toth EE Ihász F Szabo RR-B. Physical activity and psychological resilience in older adults: a systematic review of the literature. J Aging Phys Act. (2024) 32:276–86. doi: 10.1123/japa.2022-0427, PMID: 37699587

[39] Monnaatsie M Biddle SJH Kolbe-Alexander T. Feasibility of ecological momentary assessment in measuring physical activity and sedentary behaviour in shift and non-shift workers. J Act Sedent Sleep Behav. (2024) 3:24. doi: 10.1186/s44167-024-00063-7 PMC11960382 40217510

[40] Cheng Y Wang JE Rosenberg MW. Spatial access to residential care resources in Beijing, China. Int J Health Geogr. (2012) 11:32. doi: 10.1186/1476-072X-11-32, PMID: 22877360 PMC3543173

[41] Yen IH Michael YL Perdue L. Neighborhood environment in studies of health of older adults: a systematic review. Am J Prev Med. (2009) 37:455–63. doi: 10.1016/j.amepre.2009.06.022, PMID: 19840702 PMC2785463

[42] Gong Y Palmer S Gallacher J Marsden T Fone D. A systematic review of the relationship between objective measurements of the urban environment and psychological distress. Environ Int. (2016) 96:48–57. doi: 10.1016/j.envint.2016.08.019, PMID: 27599349

[43] Development CMoHaU-R. Urban residential area planning and design standard (Gb 50180-2018). Beijing, China: China Architecture & Building Press. (2018).

[44] Hoogendijk EO Deeg DJH Poppelaars J Marleen VDH Broese VG Marjolein I. The longitudinal aging study Amsterdam: cohort update 2016 and major findings. Eur J Epidemiol. (2016) 31:927–45. doi: 10.1007/s10654-016-0192-0, PMID: 27544533 PMC5010587

[45] Rajasi RS Mathew T Nujum Z Anish TS Lawrence T. Quality of life and sociodemographic factors associated with poor quality of life in elderly women in Thiruvananthapuram, Kerala. Indian J Public Health. (2016) 60:210–5. doi: 10.4103/0019-557X.189016, PMID: 27561400

[46] Fernández-Mayoralas G Rojo-Pérez F Martínez-Martín P Prieto-Flores ME Forjaz MJ. Active ageing and quality of life: factors associated with participation in leisure activities among institutionalized older adults, with and without dementia. Aging Ment Health. (2015) 19:1031–41. doi: 10.1080/13607863.2014.996734, PMID: 25584744

[47] Gottschalk S Knig HH Werner C Fleiner T Thiel C Büchele G. Association between physical activity and costs in very mild to moderately frail community-dwelling older adults: a cross-sectional study. BMC Public Health. (2024) 24:1–9. doi: 10.1186/s12889-024-20253-x, PMID: 39379954 PMC11462734

[48] Ferrucci L Cooper R Shardell M Simonsick EM Schrack JA Kuh D. Age-related change in mobility: perspectives from life course epidemiology and Geroscience. J Gerontol. (2016) 71:1184–94. doi: 10.1093/gerona/glw043, PMID: 26975983 PMC4978365

[49] Goran M Micheline W Michiel H. Short-and long-term health consequences of sleep disruption. Nat Sci Sleep. (2017) 9:151–61. doi: 10.2147/NSS.S134864, PMID: 28579842 PMC5449130

[50] Xing L Zhengwei J Jiaxin T Lei W Helang H Zhifeng W. Study on anti-aging factors and patterns of dietary types and structure of middle-aged and elderly people. Chongqing Med J. (2022) 51:3421–7. doi: 10.3969/j.issn.1671-8348.2022.20.01

[51] Mao D Li G Liang M Wang S Ren X. Dietary patterns and multiple chronic diseases in older adults. Nutr Metabol. (2024) 21:36. doi: 10.1186/s12986-024-00814-y, PMID: 38915027 PMC11194917

[52] Pan CW Wang X Ma Q Sun HP Xu Y Wang P. Cognitive dysfunction and health-related quality of life among older Chinese. Sci Rep. (2015) 5:17301. doi: 10.1038/srep17301, PMID: 26601612 PMC4658548

[53] Lopez J Perez-Rojo G Noriega C Sánchez-Cabaco A Sitges E Bonete B. Quality-of-life in older adults: its association with emotional distress and psychological wellbeing. BMC Geriatr. (2024) 24:1–6. doi: 10.1186/s12877-024-05401-7, PMID: 39385087 PMC11465940

[54] Padmanabhanunni A Pretorius TB Isaacs SA. Satisfied with life? The protective function of life satisfaction in the relationship between perceived stress and negative mental health outcomes. Int J Environ Res Public Health. (2023) 20:11. doi: 10.3390/ijerph20186777, PMID: 37754636 PMC10530804

[55] Macleod S Musich S Hawkins K Alsgaard K Wicker ER. The impact of resilience among older adults. Geriatr Nurs. (2016) 37:266–72. doi: 10.1016/j.gerinurse.2016.02.014, PMID: 27055911

[56] Lance CE Butts MM Michels LC. The sources of four commonly reported cutoff criteria: what did they really say? Organ Res Methods. (2006) 9:202–20. doi: 10.1177/1094428105284919

[57] Cohen J. Statistical power analysis for the behavioral sciences (2nd Ed): statistical power analysis for the behavioral sciences. 2nd edn. Hillsdale, New Jersey (Hillsdale, NJ): Lawrence Erlbaum Associates. (1988).

[58] Kaiser HF. An index of factorial simplicity. Psychometrika. (1974) 39:31–6. doi: 10.1007/BF02291575

[59] Williams B Brown T Onsman A. Exploratory factor analysis: a five-step guide for novices. Austral J Paramed. (2010) 8:1–13. doi: 10.33151/ajp.8.3.93

[60] Bronfenbrenner U. The ecology of human development. Cambridge, MA: Harvard University (1979).

[61] Mcleroy KR Bibeau D Steckler A Glanz K. An ecological perspective on health promotion programs. Health Educ Q. (1988) 15:351–77. doi: 10.1177/109019818801500401, PMID: 3068205

[62] Gallardo-Peralta LP Raymond M Gálvez-Nieto JL. Ageing in context: an ecological model to understand social participation among indigenous adults in Chile. Res Aging. (2023) 45:332–46. doi: 10.1177/01640275221108502, PMID: 35698297

[63] Engelen L Rahmann M De Jong E. Design for Healthy Ageing—the relationship between design, well-being, and quality of life: a review. Build Res Inform. (2022) 50:19–35. doi: 10.1080/09613218.2021.1984867, PMID: 40083416

